# Genome sequence of the flexirubin-pigmented soil bacterium *Niabella soli* type strain (JS13-8^T^)

**DOI:** 10.4056/sigs.3117229

**Published:** 2012-12-04

**Authors:** Iain Anderson, Christine Munk, Alla Lapidus, Matt Nolan, Susan Lucas, Hope Tice, Tijana Glavina Del Rio, Jan-Fang Cheng, Cliff Han, Roxanne Tapia, Lynne Goodwin, Sam Pitluck, Konstantinos Liolios, Konstantinos Mavromatis, Ioanna Pagani, Natalia Mikhailova, Amrita Pati, Amy Chen, Krishna Palaniappan, Miriam Land, Manfred Rohde, Brian J. Tindall, Markus Göker, John C. Detter, Tanja Woyke, James Bristow, Jonathan A. Eisen, Victor Markowitz, Philip Hugenholtz, Nikos C. Kyrpides, Hans-Peter Klenk, Natalia Ivanova

**Affiliations:** 1 DOE Joint Genome Institute, Walnut Creek, California, USA; 2 Los Alamos National Laboratory, Bioscience Division, Los Alamos, New Mexico, USA; 3 Biological Data Management and Technology Center, Lawrence Berkeley National Laboratory, Berkeley, California, USA; 4 Oak Ridge National Laboratory, Oak Ridge, Tennessee, USA; 5 HZI – Helmholtz Centre for Infection Research, Braunschweig, Germany; 6 Leibniz Institute DSMZ - German Collection of Microorganisms and Cell Cultures, Braunschweig, Germany; 7 University of California Davis Genome Center, Davis, California, USA; 8 Australian Centre for Ecogenomics, School of Chemistry and Molecular Biosciences, The University of Queensland, Brisbane, Australia

**Keywords:** aerobic, non-motile, Gram-negative, mesophilic, chemoorganotrophic, glycosyl hydrolases, soil, *Chitinophagaceae*, GEBA

## Abstract

*Niabella soli* Weon *et al*. 2008 is a member of the *Chitinophagaceae*, a family within the class *Sphingobacteriia* that is poorly characterized at the genome level, thus far. *N. soli* strain JS13-8^T^ is of interest for its ability to produce a variety of glycosyl hydrolases. The genome of *N. soli* strain JS13-8^T^ is only the second genome sequence of a type strain from the family *Chitinophagaceae* to be published, and the first one from the genus *Niabella.* Here we describe the features of this organism, together with the complete genome sequence and annotation. The 4,697,343 bp long chromosome with its 3,931 protein-coding and 49 RNA genes is a part of the *** G****enomic* ***E****ncyclopedia of* ***Bacteria**** and* ***Archaea***** project.

## Introduction

Strain JS13-8^T^ (= DSM 19437 = KACC 12604) is the type strain of the species *Niabella soli* [1], one out of five species in the genus *Niabella* [2]. The strain was originally isolated from soil sampled from Jeju Island, Republic of Korea [1]. The genus name was derived from the arbitrary word NIAB, National Institute of Agricultural Biotechnology, where taxonomic studies of this organism were conducted [3]; the species epithet was derived from the Latin word *soli*, of soil [1]. Strain JS13-8^T^ was found to assimilate several mono- and disaccharides and to produce numerous glycosyl hydrolases [1]. There are no PubMed records that document the use of the strain for any biotechnological studies; only comparative analyses performed for the description of later members of the genus *Niabella* are recorded. Here we present a summary classification and a set of features for *N. soli* JS13-8^T^, together with the description of the genomic sequencing and annotation.

## Classification and features

**Figure 1:**
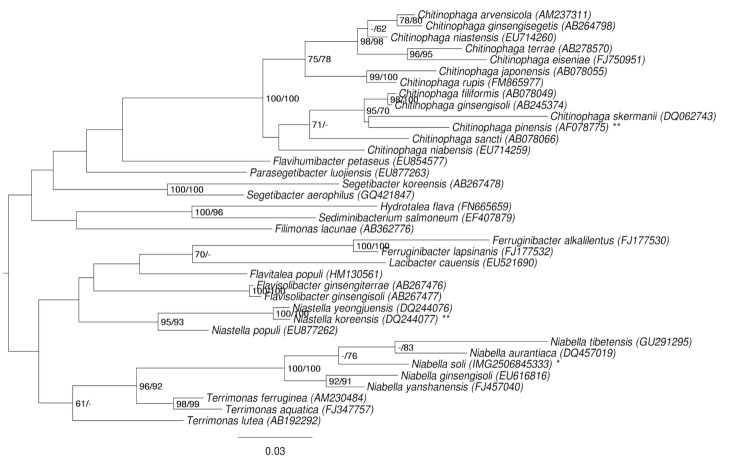
Phylogenetic tree highlighting the position of *N. soli* relative to the type strains of the other species within the family *Chitinophagaceae* except for the genera *Balneola* and *Gracilimonas*. The tree was inferred from 1,395 aligned characters [8,9] of the 16S rRNA gene sequence under the maximum likelihood (ML) criterion [10]. Rooting was done initially using the midpoint method [11] and then checked for its agreement with the current classification (Table 1). The branches are scaled in terms of the expected number of substitutions per site. Numbers adjacent to the branches are support values from 950 ML bootstrap replicates [12] (left) and from 1,000 maximum-parsimony bootstrap replicates [13] (right) if larger than 60%. Lineages with type strain genome sequencing projects registered in GOLD [14] are labeled with one asterisk, those also listed as 'Complete and Published' with two asterisks [15] (for *Niastella koreensis* see CP003178).

A representative genomic 16S rRNA sequence of *N. soli* JS13-8^T^ was compared using NCBI BLAST [4,5] under default settings (e.g., considering only the high-scoring segment pairs (HSPs) from the best 250 hits) with the most recent release of the Greengenes database [6]. The relative frequencies of taxa and keywords (reduced to their stem [7]) were determined, weighted by BLAST scores. The most frequently occurring genera were *Niabella* (34.8%), *Terrimonas* (21.0%), *Flavobacterium* (14.9%), *'Niablella'* (8.5%; an apparent misspelling of *Niabella*) and *Niastella* (8.2%) (13 hits in total). Regarding the single hit to sequences from members of the species, the average identity within HSPs was 99.7%, whereas the average coverage by HSPs was 96.8%. Among all other species, the one yielding the highest score was *'Niablella koreensis'* (DQ457019; again a misnomer, see Figure 1), which corresponded to an identity of 95.1% and an HSP coverage of 99.9%. (Note that the Greengenes database uses the INSDC (= EMBL/NCBI/DDBJ) annotation, which is not an authoritative source for nomenclature or classification.) The highest-scoring environmental sequence was JF167633 ('skin antecubital fossa clone ncd2016g05c1'), which showed an identity of 95.3% and an HSP coverage of 95.7%. The most frequently occurring keywords within the labels of all environmental samples which yielded hits were 'sludg' (3.6%), 'activ' (2.6%), 'skin' (2.3%), 'wast' (1.8%) and 'soil' (1.8%) (236 hits in total) and reveal no deeper insight into the usual habitat of close relatives of the strain. Environmental samples which yielded hits of a higher score than the highest scoring species were not found, indicating that *N. soli* itself is rarely found in environmental screenings.

**Table 1:** Classification and general features of *N. soli* JS13-8^T^ according to the MIGS recommendations [16], List of Prokaryotic names with Standing in Nomenclature [17] and the Names for Life database [2].

**MIGS ID**	**Property**	**Term**	**Evidence code**
	Current classification	Domain *Bacteria*	TAS [18]
		Phylum *Bacteroidetes*	TAS [19,20]
		Class *Sphingobacteriia*	TAS [19,21]
		Order *Sphingobacteriales*	TAS [19,22]
		Family *Chitinophagaceae*	TAS [23,24]
		Genus *Niabella*	TAS [3,23,25]
		Species *Niabella soli*	TAS [1]
		Type-strain JS13-8	TAS [1]
	Gram stain	negative	TAS [1]
	Cell shape	short rods	TAS [1]
	Motility	non-motile	TAS [1]
	Sporulation	non-sporulating	NAS
	Temperature range	mesophile, 15-35°C	TAS [1]
	Optimum temperature	30°C	TAS [1]
	Salinity	0-1% NaCl (w/v)	TAS [3]
MIGS-22	Oxygen requirement	aerobe	TAS [1]
	Carbon source	mono- and polysaccharides	TAS [1]
	Energy metabolism	chemoorganotroph	TAS [1]
MIGS-6	Habitat	soil	TAS [1]
MIGS-15	Biotic relationship	free living	TAS [1]
MIGS-14	Pathogenicity	none	NAS
	Biosafety level	1	TAS [26]
MIGS-23.1	Isolation	soil sample	TAS [1]
MIGS-4	Geographic location	Jeju Island, Republic of Korea	TAS [1]
MIGS-5	Sample collection time	not reported	
MIGS-4.1	Latitude	33.37	TAS [1]
Longitude	MIGS-4.2	126.566	TAS [1]
MIGS-4.3	Depth	not reported	
MIGS-4.4	Altitude	not reported	

Evidence codes - TAS: Traceable Author Statement (i.e., a direct report exists in the literature); NAS: Non-traceable Author Statement (i.e., not directly observed for the living, isolated sample, but based on a generally accepted property for the species, or anecdotal evidence). Evidence codes are from the Gene Ontology project [27].

Figure 1 shows the phylogenetic neighborhood of *N. soli* in a 16S rRNA based tree. The sequences of the two 16S rRNA gene copies in the genome differ from each other by one nucleotide, and differ by up to one nucleotide from the previously published 16S rRNA sequence (EF592608), which contains three ambiguous base calls.

In a preliminary phylogenetic analysis of the 16S rRNA sequences from the family, we observed that two genera, *Balneola* and *Gracilimonas*, listed as belonging to *Chitinophagaceae* by [17,28,29], formed the root of the tree and were separated from the remaining taxa by quite long branches. For this reason, they were omitted from the analysis described above, and a second phylogenetic analysis involving the type species of the type genera of all families within the phylum *Bacteroidetes* was conducted, either unconstrained or constrained for the monophyly of all families [30]. The alignment (inferred and filtered as described above) contained 17 operational taxonomic units and 1,384 characters. The best ML tree found had a log likelihood of -12,076.19, whereas the best trees found under the constraint had a log likelihood of -12,132.94. The constrained tree was significantly worse than the globally best one in the Shimodaira-Hasegawa test as implemented in RAxML [10] (α = 0.01). The bestMP trees found had a score of 2,432, whereas the best constrained tree found had a score of 2,485 and was significantly worse in the Kishino-Hasegawa test as implemented in PAUP* [13] (α = 0.01). (See, e.g. chapter 21 in [31] for an in-depth description of such paired-site tests.) This confirms our view that *Balneola* and *Gracilimonas* are misplaced as members of *Chitinophagaceae* (as all other families were represented by a single taxon only, *Chitinophagaceae* is the only family that might have caused conflict in this setting). *Chitinophagaceae* should thus be regarded to only contain the genera listed by [23] together with the more recently published genus *Flavitalea* [28].

**Figure 2:**
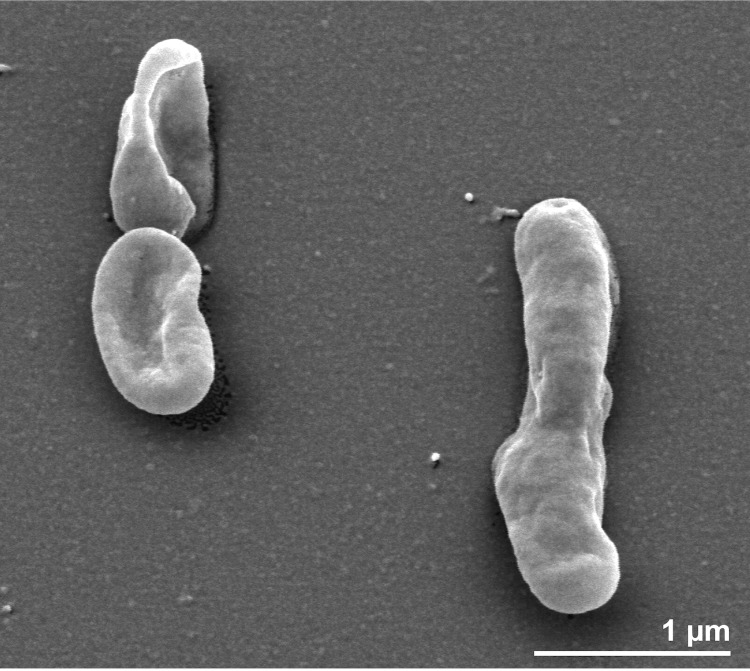
Scanning electron micrograph of *N. soli* JS13-8^T^

*N. soli* JS13-8^T^ is a Gram-negative and non-motile aerobic bacterium [1]. Cells are short rods 0.8-1.4 μm long and with a diameter of 0.5-0.7 μm ([1], Figure 2). Colonies are dark yellow due to the pigment flexirubin [1]. Growth was observed between 15°C and 35°C with an optimum at 30°C [1]. The pH range for growth was 5.0-8.0 with 6.0-7.0 as the optimum [1]. The salinity range for growth was 0-1% NaCl [3]. *N. soli* JS13-8^T^ grows on several monosaccharides, disaccharides, gluconate, and D-mannitol [1]. It produces numerous glycosyl hydrolases including α-galactosidase, β-galactosidase, β-glucuronidase, α-glucosidase, β-glucosidase, N-acetyl-β-glucosaminidase, α-mannosidase, and α-fucosidase [1]. However it did not hydrolyze starch, chitin, or carboxymethylcellulose [1].

### Chemotaxonomy

The major respiratory quinone found in *N. soli* JS13-8^T^ was MK-7, and the major fatty acids identified were *iso*-C_15:0_ (29.2%), *iso*-C_15:1 G_ (18.4%), *iso*-C_17:0 3-OH_ (11.8%), and summed feature 3 (11.1%), which is generally reported to include *iso*-C_15:0 2-OH_ and/or C_16:1 ω7c_, although careful examination of the MIDI fatty acid reports generally allow a more precise identification [1]. Smaller amounts of *anteiso*-C_15 : 0_ (1.2%), *iso*-C_15:0 3-OH_ (2.2%), C_16:0_ (6.8%), C_16:0 2-OH_ (1.3%), C_16:0 3-OH_ (2.2%), C_18:0_ (3.8%), C_18:1 ω7c_ (1.5%), C_18:1 ω9c_ (1.0%), Summed feature 5 (comprising *anteiso*-C_18:0_ and/or C_18:2 ω6,9c_ 3.4%) and an unknown peak with an equivalent chain length of 13.565 (1.1%) were also detected. The presence of major amounts of branched chain saturated and unsaturated fatty acids, together with significant amounts of 3-OH and 2-OH fatty acids is characteristic of members of this evolutionary group and also points to the presence of characteristic lipids, for which data is missing from this strain.

## Genome sequencing and annotation

### Genome project history

This organism was selected for sequencing on the basis of its phylogenetic position [32], and is part of the *** G****enomic* ***E****ncyclopedia of* ***Bacteria**** and* ***Archaea***** project [33]. The genome project is deposited in the Genomes On Line Database [14] and the complete genome sequence is deposited in GenBank. Sequencing, finishing and annotation were performed by the DOE Joint Genome Institute (JGI). A summary of the project information is shown in Table 2.

**Table 2:** Genome sequencing project information

**MIGS ID**	**Property**	**Term**
MIGS-31	Finishing quality	Finished
MIGS-28	Libraries used	Five genomic libraries: two 454 pyrosequence standard libraries, two 454 PE library (13 kb and 20 kb insert size), one Illumina library
MIGS-29	Sequencing platforms	Illumina GAii, 454 GS FLX Titanium
MIGS-31.2	Sequencing coverage	113.0 × Illumina; 23.6 × pyrosequence
MIGS-30	Assemblers	Newbler version 2.3, Velvet version 1.0.13, phrap version SPS - 4.24
MIGS-32	Gene calling method	Prodigal
	INSDC ID	AGSA00000000
	GenBank Date of Release	January 19, 2012
	GOLD ID	Gi04680
	NCBI project ID	61269
	Database: IMG	2506783006
MIGS-13	Source material identifier	DSM 19437
	Project relevance	Tree of Life, GEBA

### Growth conditions and DNA isolation

*N. soli* strain JS13-8^T^, DSM 19437, was grown in DSMZ medium 830 (R2A medium) [34] at 37°C. DNA was isolated from 0.5-1 g of cell paste using MasterPure Gram-positive DNA purification kit (Epicentre MGP04100) following the standard protocol as recommended by the manufacturer with modification st/DL for cell lysis as described in Wu *et al*. 2009 [33]. DNA is available through the DNA Bank Network [35].

### Genome sequencing and assembly

The genome was sequenced using a combination of Illumina and 454 sequencing platforms. All general aspects of library construction and sequencing can be found at the JGI website [36]. Pyrosequencing reads were assembled using the Newbler assembler (Roche). The initial Newbler assembly consisting of 15 contigs in one scaffold was converted into a phrap [37] assembly by making fake reads from the consensus, to collect the read pairs in the 454 paired end library. Illumina GAii sequencing data (1,116.9 Mb) was assembled with Velvet [38] and the consensus sequences were shredded into 1.5 kb overlapped fake reads and assembled together with the 454 data. The 454 draft assembly was based on 158.8 Mb of 454 draft data and all of the 454 paired end data. Newbler parameters are -consed -a 50 -l 350 -g -m -ml 20. The Phred/Phrap/Consed software package [37] was used for sequence assembly and quality assessment in the subsequent finishing process. After the shotgun stage, reads were assembled with parallel phrap (High Performance Software, LLC). Possible mis-assemblies were corrected with gapResolution [36], Dupfinisher [39], or sequencing cloned bridging PCR fragments with subcloning. Gaps between contigs were closed by editing in Consed, by PCR and by Bubble PCR primer walks (J.-F. Chang, unpublished). A total of 45 additional reactions were necessary to close gaps and to raise the quality of the finished sequence. Illumina reads were also used to correct potential base errors and increase consensus quality using a software Polisher developed at JGI [40]. The error rate of the completed genome sequence is less than 1 in 100,000. Together, the combination of the Illumina and 454 sequencing platforms provided 136.6 × coverage of the genome. The final assembly contained 354,991 pyrosequence and 14,750,629 Illumina reads.

### Genome annotation

Genes were identified using Prodigal [41] as part of the Oak Ridge National Laboratory genome annotation pipeline, followed by a round of manual curation using the JGI GenePRIMP pipeline [42]. The predicted CDSs were translated and used to search the National Center for Biotechnology Information (NCBI) non-redundant database, UniProt, TIGRFam, Pfam, PRIAM, KEGG, COG, and InterPro databases. These data sources were combined to assert a product description for each predicted protein. Non-coding genes and miscellaneous features were predicted using tRNAscan-SE [43], RNAmmer [44], Rfam [45], TMHMM [46], and signalP [47].

## Genome properties

**Figure 3:**
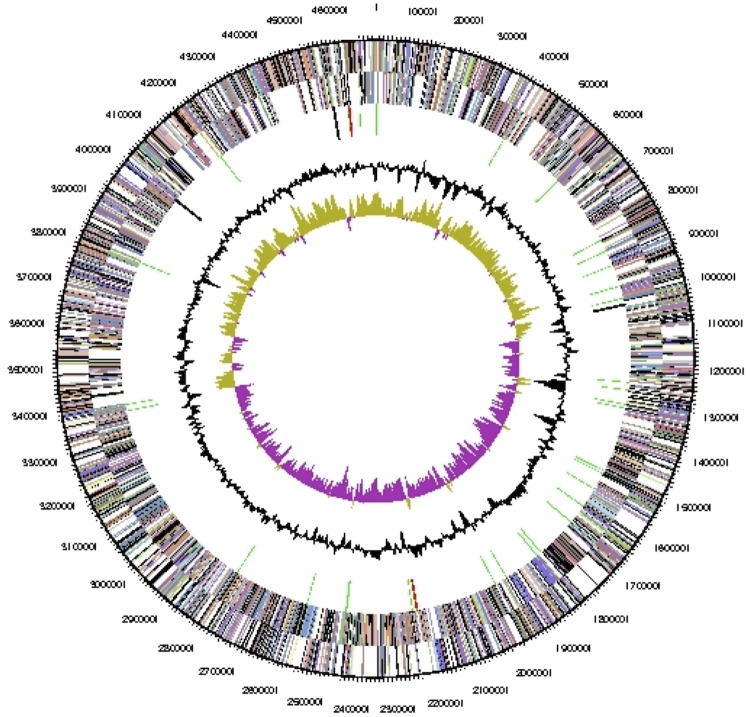
Graphical map of the chromosome. From outside to the center: Genes on forward strand (colored by COG categories), Genes on reverse strand (colored by COG categories), RNA genes (tRNAs green, rRNAs red, other RNAs black), GC content (black), GC skew (purple/olive).

The genome consists of one circular chromosome of 4,697,343 bp length with a 45.2% G+C content (Table 3 and Figure 3). Of the 3,932 genes predicted, 3,882 were protein-coding genes, and 49 RNAs; 34 pseudogenes were also identified. The majority of the protein-coding genes (71.9%) were assigned a putative function while the remaining ones were annotated as hypothetical proteins. The distribution of genes into COGs functional categories is presented in Table 4.

**Table 3:** Genome Statistics

**Attribute**	Value	% of Total
Genome size (bp)	4,697,343	100.00%
DNA coding region (bp)	4,154,623	88.45%
DNA G+C content (bp)	2,124,959	45.24%
Number of replicons	1	
Extrachromosomal elements	0	
Total genes	3,931	100.00%
RNA genes	6	0.15%
rRNA operons	2	
tRNA genes	49	1.25%
Protein-coding genes	3,882	98.75%
Pseudo genes	34	0.86%
Genes with function prediction (proteins)	2,827	71.92%
Genes in paralog clusters	1,833	46.63%
Genes assigned to COGs	2,734	69.55%
Genes assigned Pfam domains	2,915	74.15%
Genes with signal peptides	1,273	32.38%
Genes with transmembrane helices	924	23.51%
CRISPR repeats	1	

**Table 4:** Number of genes associated with the general COG functional categories

**Code**	**value**	**%age**	**Description**
J	156	5.2	Translation, ribosomal structure and biogenesis
A	0	0.0	RNA processing and modification
K	204	6.8	Transcription
L	115	3.9	Replication, recombination and repair
B	0	0.0	Chromatin structure and dynamics
D	21	0.7	Cell cycle control, cell division, chromosome partitioning
Y	0	0.0	Nuclear structure
V	68	2.3	Defense mechanisms
T	132	4.4	Signal transduction mechanisms
M	239	8.0	Cell wall/membrane biogenesis
N	5	0.2	Cell motility
Z	1	0.0	Cytoskeleton
W	0	0.0	Extracellular structures
U	49	1.6	Intracellular trafficking and secretion, and vesicular transport
O	123	4.1	Posttranslational modification, protein turnover, chaperones
C	151	5.1	Energy production and conversion
G	293	9.8	Carbohydrate transport and metabolism
E	231	7.7	Amino acid transport and metabolism
F	69	2.3	Nucleotide transport and metabolism
H	143	4.8	Coenzyme transport and metabolism
I	104	3.5	Lipid transport and metabolism
P	178	6.0	Inorganic ion transport and metabolism
Q	51	1.7	Secondary metabolites biosynthesis, transport and catabolism
R	383	12.8	General function prediction only
S	268	9.0	Function unknown
-	1,197	30.5	Not in COGs

## Insights into the genome sequence

Two other complete genomes are available in GenBank from the family *Chitinophagaceae* – *Chitinophaga pinensis* [15] and *N. koreensis* (unpublished) – and the permanent draft genome of *Sediminibacterium sp.* OR43 is available from the IMG/GEBA website [48]. Of these three organisms, *N. soli* is most closely related to *N. koreensis* (Figure 1). The genome size of *N. soli* is much smaller than those of *N. koreensis* and *C. pinensis* (9.0-9.1 Mbp) but larger than that of *Sediminibacterium sp.* OR43 (3.8 Mbp). Using the genome-to-genome distance calculator [49,50] version 2.0 revealed that 83.72% of all positions within HSPs are identical between the type-strain genomes of *N. soli* and *C. pinensis*, which corresponds to a DNA-DNA hybridization value of 26.60±2.42%. For *N. koreensis*, these values were 78.29% and 20.20±2.31%, respectively.

A major feature of the previously sequenced genomes from this family is the presence of large numbers of glycosyl hydrolases. *N. koreensis* has 228 glycosyl hydrolases, while *C. pinensis* has 187 [51]. We analyzed the genomes of *N. soli* and strain OR43 and found that they encode 164 and 86 glycosyl hydrolases, respectively. When viewed as a percentage of the total protein-coding sequences, glycosyl hydrolases constitute 4.2% of the *N. soli* genome and 3.1% of the *N. koreensis* genome. In the *C. pinensis* and OR43 genomes, glycosyl hydrolases account for 2.6% of the protein-coding genes. Thus *N. soli* has the highest density of glycosyl hydrolases in this family examined to date. In addition *N. koreensis* has 28 polysaccharide lyases while *C. pinensis* has only six [51]. We found that *N. soli* has 15 polysaccharide lyases and OR43 has only two. Thus *N. soli* also has a substantial number of polysaccharide lyases in addition to glycosyl hydrolases.

Of the glycosyl hydrolase families with many members in *N. soli*, some are also prevalent in *N. koreensis* and *C. pinensis*, for example families GH2, GH28, GH29, GH43, and GH78. However, there are GH families in which *N. soli* has a greater number of members than the genomes from other *Chitinophagaceae* – GH20 and GH106. *N. soli* also has enzymes from GH116 and GH123, which are not found in the other three genomes. There is also one GH family (GH92) for which *N. soli* has only two members, while *N. koreensis* and *C. pinensis* have 10 and 9, respectively.

In *Bacteroides thetaiotaomicron*, the SusC and SusD outer membrane proteins are required for starch utilization [52] and the *B. thetaiotaomicron* genome contains many proteins related to SusC and SusD [53]. The genomes from the family *Chitinophagaceae* also contain large numbers of these proteins. *N. soli* has 60 SusC family and 50 SusD family proteins, which is about half as many as in the larger *N. koreensis* and *C. pinensis* genomes.

The *Chitinophagaceae* appear to rely mainly on symporters for sugar transport. Only two sugar ABC transporters were found in *N. soli*, one in *N. koreensis*, and none in the other two genomes. The phosphotransferase system is not found in any of the four genomes. In contrast *N. soli* has 23 sugar symporters, *N. koreensis* has 27, *C. pinensis* has 14, and OR43 has 12. The sugar symporters belong to several families, with the most prevalent being the Major Facilitator Superfamily (TC 2.A.1) and the Solute:Sodium Symporter Family (TC 2.A.21).
